# Precision nomenclature for the new genomics

**DOI:** 10.1093/gigascience/giz086

**Published:** 2019-08-22

**Authors:** Harris A Lewin, Jennifer A Marshall Graves, Oliver A Ryder, Alexander S Graphodatsky, Stephen J O'Brien

**Affiliations:** 1 Department of Evolution and Ecology and the UC Davis Genome Center, 4321 Genome and Biomedical Sciences Facility, University of California, Davis, CA 95616, USA; 2 Department of Ecology, Environment and Evolution, School of Life Sciences, La Trobe University, Kingsbury Drive, Bundoora , Victoria 3086, Australia; 3 San Diego Zoo Institute for Conservation Research, 15600 San Pasqual Valley Road, Escondido, CA 92027, USA; 4 Institute of Molecular and Cellular Biology, SB RAS, Novosibirsk 630090, Russia; 5 Novosibirsk State University, Novosibirsk 630090, Russia; 6 Theodosius Dobzhansky Center for Genome Bioinformatics, St. Petersburg State University, St. Petersburg 194044, Russia; 7 Halmos College of Natural Sciences and Oceanography, Nova Southeastern University, Ft. Lauderdale, FL 33004, USA

## Abstract

The confluence of two scientific disciplines may lead to nomenclature conflicts that require new terms while respecting historical definitions. This is the situation with the current state of cytology and genomics, which offer examples of distinct nomenclature and vocabularies that require reconciliation. In this article, we propose the new terms *C-scaffold* (for chromosome-scale assemblies of sequenced DNA fragments, commonly named *scaffolds*) and *scaffotype* (the resulting collection of C-scaffolds that represent an organism's genome). This nomenclature avoids conflict with the historical definitions of the terms *chromosome* (a microscopic body made of DNA and protein) and *karyotype* (the collection of images of all chromosomes of an organism or species). As large-scale sequencing projects progress, adoption of this nomenclature will assist end users to properly classify genome assemblies, thus facilitating genomic analysis.

## Background

Long-read DNA sequencing, chromatin conformation capture techniques, and optical mapping methods now make it possible to approach a complete, contiguous, phased, and ordered representation of the DNA sequence of chromosomes. Chromosome-level assembly is fast becoming the gold standard to be applied to *de novo* whole-genome sequencing [1]. To avoid misrepresentation and confusion in the scientific community, there is an urgent need to adopt nomenclature for these assemblies that is consistent with well-accepted definitions used in genomics and cytology.

A reference-quality chromosome-scale whole-genome sequence can now be produced *de novo* for a tiny fraction of the original cost of sequencing the human genome. Several consortia are therefore actively striving to achieve near-complete and accurately ordered genome sequences using the newly available approaches. For example, the G10K-Vertebrate Genomes Project recently released new chromosome-scale assemblies of 14 vertebrate species [2] and is projecting hundreds more chromosome-scale assemblies in 2019. The Earth BioGenome Project [3] aims to produce more than 9000 reference-quality eukaryote genomes. Reference-quality genome sequences for large numbers of eukaryotic taxa will greatly accelerate our understanding of evolution, adaptation, and speciation.

It has been recognized that a good physical gene map greatly facilitates assembly of incomplete sequences, allowing a correct chromosomal ordering of assembly scaffolds [4]. However, new sequencing and assembly methods now make it possible to build chromosome-scale scaffolds in the absence of a map or even a cytological description. This is just as well, for genome sequencing is fast outpacing cytological studies. So far, cytological karyotypes have been complied for only 1160 of the 5500 named mammal species [5] and even fewer for other vertebrates and invertebrates. This means that there will soon be species for which a fully sequenced genome, but no karyotype, is available.

## Nomenclature for Assemblies of DNA Fragments

Names applied in the literature to longer assemblies of sequenced DNA fragments include contig, scaffold, and superscaffold (see Text Box). The International Nucleotide Sequence Database Collaboration that involves major sequence archives now designates certain scaffold-based assemblies to be given the designation “chromosome level” and super-scaffolds to be named simply “chromosomes,” even in the absence of physical mapping data (e.g., pale spear-nosed bat: [6] and greater horseshoe bat: [7]).

This contributes to substantial confusion and frequent misunderstanding. Imprecise terminology for these large scaffolds presents an issue for users of the databases, who need to quantify how complete a deposited genome sequence actually is, as well as to distinguish assemblies that have comprehensive physical assignments to chromosomes from those that are only scaffold based.

Perhaps even more important, it is now clear that the non-DNA components of chromosomes, particularly modified histones, exercise profound influence over chromosome conformation and the expression of genes in development, as well as change as the result of environmental influences (epigenetic variation) [8, 9]. In addition, the position of genes on the chromosome with respect to its centromere, telomeres, and heterochromatin, as well as the position of a chromosome in the interphase nucleus of the cell, can affect expression. Thus, studies of chromosome evolution, the evolution of gene regulation by changes in chromosome conformation, and epigenetics all require or benefit from distinguishing scaffold-based versus chromosome-anchored assemblies versus functional chromosomes.

## Proposed Nomenclature

There are, therefore, different ways to describe elements of a species’ genome. Descriptors sometimes overlap but must often be distinguished. We define established terms in the Text Box and propose new specific terms for chromosome-scale assemblies in order to:

1. avoid conflict with established terminology,
2. avoid problems downstream for species with a scaffold-only-based representation of its chromosomes and for which no karyotype is available, and
3. make clear to end users of the new assemblies exactly what is being described.

We justify the distinction of the new terms as follows. The term *chromosome* (from Greek roots for “colored body”) was coined in 1888 [10]. A century ago chromosomes were found to contain the genes of an organism. In eukaryotes, chromosomes are widely recognized as DNA-protein complexes housing a single linear DNA molecule that bears a linear array of genes. Chromosomes during cell interphase exist as long threadlike chromatin in the nucleus, but during mitosis, they contract into rod-shaped bodies that can be visualized using microscopy. Thus, the term *chromosome* has a clear definition that has been in use for 130 years.

A DNA scaffold, no matter how large or complete, can never be a chromosome. It is a simply an abbreviated digital script of the linear DNA sequence on a chromosome. For assignment of DNA sequence to a chromosome, it must be physically mapped by methods such fluorescence *in situ* hybridization or by inference if a scaffold includes DNA marker sequences that were previously mapped.

DNA sequencing technology has not yet advanced to produce a single molecule that represents an entire chromosome in any large multicellular eukaryote. However, in the future, a new generation of long-read technology may yield contiguous fragments that span the entire chromosome length, most likely for eukaryotes with small genomes. Ungapped contiguous overlapping fragments of DNA sequence are termed *contigs*, and for consistency with the proposed nomenclature for chromosome-scale scaffolds (see below), we propose the term *chromosome-scale contig*, or *C-contig*, to define an ungapped assembly of DNA fragments that spans an entire chromosome (autosome or sex chromosome) or the circular genome of mitochondria. Such contigs have been produced for bacterial chromosomes and chromosomes of yeasts and fungi (e.g., S*accharomyces cerevisiae, Kluyveromyces lactis*, and *Candida dubliniensis*).

We propose to name a scaffold or super-scaffold that appears to span the full length of a chromosome or a chromosome arm as a *chromosome-scale scaffold*, abbreviated as *C-scaffold*. Even with the powerful technologies available today, for the vast majority of eukaryotic species, the largest scaffolds have appreciable sequence gaps and are frequently missing highly repetitive regions such as centromeres and telomeres. Thus, chromosome arms will often be the largest units that can be completely represented by a *C-scaffold*.

In the same way, the term *karyotype* (derived from the Greek word *karyon*, meaning “nucleus”) has been used for a century to describe the complete chromosome complement of a cell or organism. In modern times a karyotype is represented as an ordered array (usually by descending size) of metaphase chromosomes in a photographic or diagrammatic image, with centromere position and any abnormalities noted. An ordered array of *C-scaffolds* (and/or *C-contigs*) is not the same thing and needs a new name. We propose the term *scaffotype*.

Most eukaryotic assemblies to date include more scaffolds than chromosomes or chromosome arms. Thus, it is impossible to determine exactly from the scaffolds the actual number of chromosomes of a species. We recommend that karyotypes of sequenced organisms be assessed when feasible, certainly for reference-quality genomes of representative species. In our view, the ultimate goal for an ideal genome assembly is to produce a scaffotype of C-scaffolds (or, in the future, C-contigs) that have all been assigned to physical chromosomes; thus, the scaffotype becomes a complete molecular description of a species’ linear nucleotide sequence, matched to its karyotype.

## Conclusions

Precise and accurate terminology is a requisite hallmark of good science. In biology, the definition of terms is sometimes ambiguous or misappropriated. Extensive use of jargon complicates matters even further. We believe that it is early enough on the journey of producing chromosome-scale *de novo* assemblies to implement terminology formally that accurately reflects chromosome-scale *in silico* constructs of DNA fragments without altering existing definitions that have served the genetics community so well.

**Box: Terms and definitions of cytogenetics and large sequence arrays**

**Standard Cytogenetic Definitions**

**Chromosome** (literally “colored body”): DNA and protein-containing structure in cells of eukaryotes, microscopically visible as a rod-shaped body during cell division metaphase.

**Karyotype**:  A photographic or diagrammatic image of the complete set of metaphase chromosomes in cells of an organism of a particular species.

**Standard Molecular Descriptors**

**Contigs**: Contiguous gapless stretches of DNA sequence assembled from smaller overlapping sequenced fragments.

**Scaffolds**: Computationally ordered and oriented arrays of contigs that have sequence gaps along their length.

**Super-scaffolds**: Ordered scaffolds produced by methods such as optical mapping and chromosome conformation capture technologies .

**Proposed New Terms**

**C-contig** (chromosome-scale contig): A contig that appears to span all of a chromosome arm or a complete chromosome.

**C-scaffold** (chromosome-scale scaffold): A scaffold or super-scaffold that appears to span all of a chromosome arm or a complete chromosome.

Evidence that a contig, scaffold, or super-scaffold represents a chromosome or chromosome arm can come from Hi-C data and be corroborated by optical maps.

A C-contig or C-scaffold is formally assigned to a chromosome when it is physically mapped to a known chromosome in a species having an established karyotype (e.g., using fluorescence *in situ* hybridization). For fluorescence *in situ* hybridization, we recommend that multiple included DNA markers be mapped along the length of the C-scaffold, to establish orientation.

**Scaffotype**: A set of C-scaffolds and/or C-contigs that are a representation of all the chromosomes, including sex chromosomes, of a species.

The C-scaffolds and C-contigs in a scaffotype should be numbered continuously according to descending length in the assembly.

If the C-scaffolds and C-contigs are all mapped to chromosomes, and the number of chromosomes and C-scaffolds is identical, then the scaffotype and the karyotype terms reflect equivalent representations of the complete chromosome complement of an organism or species.

## Authors’ contributions

All authors contributed equally to the manuscript. H.A.L J.A.M.G and S.J.O.

## Editor's Note

This is an ongoing discussion in the community and will be debated and discussed this month at the upcoming G10K-VGP/EBP Meeting at Rockerfeller University (27-30th August 2019). We encourage comments and feedback to aid in forming a consensus on the most suitable nomenclature for chromosome level sequence. One way to discuss these matters is via social media and annotation of this paper by hypothes.is (use the hashtag/tag #chromosomenomenclature). We hope *GigaScience* can be a forum to assist this discourse.
